# Scaffold-free 3D cell culture of primary skin fibroblasts induces profound changes of the matrisome

**DOI:** 10.1016/j.mbplus.2021.100066

**Published:** 2021-05-12

**Authors:** Bich Vu, Glauco R. Souza, Jörn Dengjel

**Affiliations:** a Department of Biology, University of Fribourg, Chemin du Musée 10, CH-1700 Fribourg, Switzerland; b Greiner Bio-One North America, Inc., 4238 Capital Drive, Monroe, NC 28110, USA

**Keywords:** ECM, extracellular matrix, MS, mass spectrometry, NHF, normal human fibroblast, SCC, squamous cell carcinoma, SILAC, stable isotope labelling by amino acids in cell culture, Skin, Fibroblast, Proteomics, Mass spectrometry, Matrigel, Matrisome, Basement membrane, Collagen, Bioprint, Magnetic, Nanoshuttle

## Abstract

The human skin has a highly developed extracellular matrix (ECM) that is vital for proper skin functioning, its 3D architecture playing a pivotal role in support and guidance of resident and invading cells. To establish relevant *in vitro* models mimicking the complex design observed *in vivo,* scaffold-based and scaffold-free 3D cell culture systems have been developed. Here we show that scaffold-free systems are well suited for the analysis of ECM protein regulation. Using quantitative mass spectrometry-based proteomics in combination with magnetic 3D bioprinting we characterize changes in the proteome of skin fibroblasts and squamous cell carcinoma cells. Transferring cells from 2D to 3D without any additional scaffold induces a profound upregulation of matrisome proteins indicating the generation of a complex, tissue-like ECM.

## Introduction

*In vitro* cultures of mammalian cells contributed invaluably to our understanding of biochemical and cell biological processes. Also in translational medicine, cell culture systems are routinely used to study pathophysiological processes as well as mechanisms-of-actions of drug candidates. However, although drug candidates are extensively studied *in vitro* as well as *in vivo* using respective animal models, more than 90% of drug candidates that enter clinical trials fail to reach administrative approval [1], [2]. One reason for this are interspecies variations that interfere with extrapolations performed using data generated in pre-clinical animal models. Often these do not recapitulate all details of human biology and impede translation of pre-clinical results. One technique that can help addressing this problem are three dimensional (3D) human cell culture systems, which often closer recapitulate *in vivo* observations compared to two dimensional (2D) cultures [3], and which are more and more employed in drug development and testing [1]. This is especially true when studying influences of the extracellular matrix (ECM), which transfers both biochemical and physical stimuli affecting virtually all aspects of cell biology: cell migration, differentiation, proliferation and viability [4], [5].

3D cell cultures can be broadly divided into scaffold-based and scaffold-free systems [6]. Whereas scaffold-based systems allow better manipulation of spheroid cultures such as position, geometry and size, they commonly raise concerns about biocompatibility and clinical application as generally non-human materials are employed to generate scaffolds. This holds true for both non-biomolecule scaffolds like polyurethane as well as biomolecule-based scaffolds like polylactic acid or collagen, which is commonly isolated from animal sources [6], [7]. A widely used scaffold in basic science is Matrigel™, a gelatinous protein mixture produced by Engelbreth-Holm-Swarm mouse sarcoma cells [8]. Due to its source, Matrigel is rich in basement membrane proteins, such as laminins beta-1 and gamma-1, collagen IV and nidogen-1 [9], and has amongst others pro-angiogentic and adipogenic properties [8]. The immense number of studies that employed Matrigel clearly argue for its usefulness. However, due to its biological activities and its purification from sarcoma cells there are also concerns that question the *in vivo* relevance of this model system and *in vitro* reproducibility [10]. Regardless which types of scaffolds are employed, all of them usually interfere with the analytical characterization of biomolecules released by embedded cells. Cells themselves can be purified from scaffolds in order to analyze e.g. their regulation on transcriptome and proteome level [11]. In contrast, deposited ECM proteins or secreted factors are difficult to purify from scaffolds due to protein-scaffold interactions. If scaffolds are protein-based themselves, such as Matrigel, they interfere with protein characterization requiring e.g. the use of metabolic labelling approaches to discriminate cell- from scaffold-derived proteins [9].

Whereas the generation of sufficient numbers of scaffold-free spheroids in a reproducible quality was initially challenging, this limitation has been successfully addressed [6], [12]. Current scaffold-free 3D cell cultures address many of the above-mentioned constraints of scaffold-based approaches, such as limited biocompatibility and potential approval for clinical use. Commonly employed scaffold-free approaches include hanging drop cultures, usage of non-adhesive wells or rotating wall vessels, and magnetic levitation and bioprinting [12], [13]. As spheroids are made up of human cells and their deposited ECM only, these approaches are also better suited for bioanalytical approaches addressing basic scientific questions, such as regulation of protein abundances due to a 3D environment. The absence of a protein-containing scaffold should facilitate especially the analysis of protein secretion and ECM deposition by mass spectrometry (MS)-based proteomics.

In the current report, we discuss the effect of magnetic 3D bioprinting using NanoShuttle™-PL on the transcriptome and proteome of primary human skin fibroblasts and epidermal squamous cell carcinoma cells and compare it to 3D cultures based on Matrigel. NanoShuttle-PL employs magnetic nanoparticles that interact electrostatically with cells rendering them magnetic and allowing manipulation of their 3D assembly using external magnetic forces. Scaffold-free spheroids can be generated within few days and are readily analyzable by proteomic approaches. We focus our analysis on the matrisome, i.e. the set ECM and ECM-associated proteins [14], particularly diverse in skin and essential for skin physiology [5]. Importantly, as described for scaffold-based approaches 3D culturing by magnetic bioprinting leads to an upregulation of matrisome proteins and proteins involved in cell-matrix contacts. Due to the absence of a scaffold a deeper coverage of the proteome is achieved, allowing more detailed insights into protein regulation.

## Materials and methods

### Cells

Primary normal human fibroblasts (NHF) were isolated from the foreskin of a circumcised 4-year-old boy after written informed consent. SCC13 is an established cancer cell line isolated from a skin squamous cell carcinoma of a 56-year-old female (https://web.expasy.org/cellosaurus/CVCL_4029).

### Standard 2D cell culture

NHF were maintained in Dulbecco's Modified Eagle Medium (DMEM) with 4.5 g/L glucose, stable glutamine, sodium pyruvate and 3.7 g/L NaHCO_3_ (PAN Biotech), supplemented with 10% foetal bovine serum (FBS, Gibco) and 1% penicillin/streptomycin (PAN Biotech). SCC13 cells were maintained in Keratinocyte Serum Free Medium (SFM) with l-Glutamine (Gibco), supplemented with 2.5 μg epidermal growth factor and 25 mg bovine pituitary extract (Gibco), as well as 1% penicillin/streptomycin. All cells were kept at 37 °C with 5% CO_2_. To passage at confluency, cells were washed with 1× phosphate-buffered saline solution (1×-PBS, PAN Biotech) and detached with 0.05% trypsin/0.02% EDTA in PBS without Ca and Mg (PAN Biotech).

### Stable isotope labelling by amino acids in cell culture (SILAC)

SILAC labelling was performed as previously described [15], [16], briefly: NHF were cultured and passaged in SILAC-DMEM with 4.5 g/L glucose, sodium pyruvate and 3.7 g/L NaHCO_3_ (PAN Biotech), supplemented with 10% dialyzed FBS (Gibco), 1% penicillin/streptomycin, 82 mg/L l-proline, 84 mg/L l-arginine HCl (Arg0) and 146 mg/L l-lysine HCl (Lys0) for light labelling (Sigma-Aldrich). For medium-heavy labelled cells, Arg0 and Lys0 were replaced by l-arginine-^13^C_6_^14^N_4_ and l-lysine-^2^H_4_ (Arg6, Lys4). For heavy labelling, l-arginine-^13^C_6_^15^N_4_ and l-lysine-^13^C_6_^15^N_2_ (Arg10, Lys8) were used.

SCC13 cells were cultured in SILAC-Keratinocyte Growth Medium 2 (KGM2) without calcium, l-arginine, and l-lysine (PromoCell), supplemented with SupplementMix containing 0.004 mL/mL bovine pituitary extract, 0.125 ng/mL epidermal growth factor, 5 μg/mL insulin, 0.33 μg/mL hydrocortisone, 0.39 μg/mL epinephrine, 10 μg/mL transferrin and 0.06 μM CaCl_2_ (PromoCell), with 1% penicillin/streptomycin. l-Lysine HCl concentrations were the same as mentioned above. For l-arginine HCl, 210 mg/L were used [17].

### 3D cell culture

Cells were first grown in standard 10 cm dishes. After 1 week of SILAC labelling for SCC13 cells and 2 weeks of labelling for NHF, all cells were transferred to a standard 6-well plate, with 1 million cells per well. Cells were magnetized using 1 µL of magnetic nanoparticle assembly (Nanoshuttle from the 6-well Bio-Assembler Kit, Greiner Bio-One, GmbH) per 10,000 cells. The day after, magnetized cells were trypsinized and transferred to a 6-well cell-repellent plate, with a magnetic concentrating drive underneath. The following day, 50 µg/mL l-ascorbic acid (Sigma) was added to all cells. Ascorbic acid was added every other day for 6 days to promote collagen synthesis. Cells grown in 2D were kept in regular 6-well plates, labelled for the same amount of time, including the addition of l-ascorbic acid.

### RNA extraction and protein purification

Cells were lysed directly on the plate using RLT buffer, supplemented with 20 mM β-mercaptoethanol. Cell lysate including ECM were scraped and harvested before RNA extraction using the RNeasy Mini Kit with QIAshredders (Qiagen). Proteins were recovered from flow through and precipitated in 100% methanol overnight at −20 °C. Proteins were resuspended in 4% SDS and protein concentration was determined using the BCA Protein Assay Kit according to manufacturer's procedures (Pierce).

### Mass spectrometry analyses

Light labelled NHF and SCC13 samples were pooled to a Super-SILAC mix to serve as spike-in [18]. NHF and SCC13 samples were then mixed separately in a ratio of 1:1:1 of light, medium and heavy samples. Samples were taken up in SDS loading buffer, followed by reduction with 1 mM DTT (VWR) for 10 min at 75 °C, and alkylation in the dark with 5.5 mM iodoacetamide (Fluka) for 10 min at room temperature. Samples were separated on 4–12% Express Plus PAGE gels (GenScript). Gel lanes were cut into 5 equal slices, which were then cut into smaller pieces. Proteins were digested by addition of 80 μL sequencing grade modified trypsin (Promega) per gel slice overnight. Peptides were extracted, concentrated using a SpeecVac vacuum concentrator (Thermo Fisher Scientific) and purified by STAGE tips as described [19].

Samples were analysed by a Q Exactive Plus mass spectrometer coupled to an EasyLC 1000 (all Thermo Fisher Scientific) essentially as described [20], briefly: peptides were fractionated on a fused silica HPLC-column tip (I.D. 75 μm, New Objective, self-packed with ReproSil-Pur 120 C18-AQ, 1.9 μm (Dr. Maisch) to a length of 20 cm) using a gradient of A (0.1% formic acid in water) and B (0.1% formic acid in 80% acetonitrile in water): peptides were loaded with 0% B with a flow rate of 600 nL/min and separated by 5%–30% B within 85 min with a flow rate of 250 nL/min. Spray voltage was set to 2.3 kV and the ion-transfer tube temperature to 250 °C; no sheath and auxiliary gas were used. The mass spectrometer was operated in the data-dependent mode; after each MS scan (mass range *m*/*z* = 370–1750; resolution: 70,000) a maximum of ten MS/MS scans were performed using a normalized collision energy of 25%, a target value of 1000 and a resolution of 17,500.

MS raw files were analysed using the MaxQuant software version 1.6.2.10 [21], with a UniProt human database from March 2016 (21,033 entries). Cysteine carbamidomethylation was set as fixed modification, whereas methionine oxidation and protein N-terminal acetylation were set as variable modifications. Trypsin/P was set as specific digestion mode, with up to three missed cleavages. Quantitation was set for triple SILAC. Peptide and protein FDRs were set to 0.01 for identification. Minimum peptide length was set to 7 and minimum unique peptide was set to 1. For protein quantification, minimum ratio count was set to 2 and only unmodified peptides, peptides with oxidized methionine or carrying N-terminal protein acetylation were used. The “match between runs” setting was used as well. Raw data are available via ProteomeXchange with identifier PXD023701 [22].

### Western blot

Protein samples were prepared by adding SDS loading buffer with 1 mM DTT for 10 min at 75 °C to whole cell lysate. Proteins were separated by SDS-PAGE prior transfer onto nitrocellulose membranes with a 0.45 µm pore size (Amersham). Wet blot was performed for 2 h 30 min at 100 V. Membranes were saturated for 1 h with 5% milk (Roth) in TBS with 0.1% Tween-20 (TBST, Sigma-Aldrich) at room temperature.

Primary antibodies Tenascin C (MAB2138, R&D Systems, rat), Collagen-VII alpha 1 (NC2-10, in house, rabbit) and Nidogen-1H-200 (sc-33141, Santa Cruz Biotech, rabbit), were diluted 1:1000 in 5% milk in TBST and used overnight at 4 °C. Membranes were washed three times with TBST, with constant shaking. Secondary antibodies Goat Anti-Rat IgG H + L HRP (31470, Thermo Fisher Scientific) and peroxidase-conjugated Goat Anti-Rabbit (Jackson 111-035-045) were diluted 1:10,000 in 5% milk in TBST and used for 1 h at room temperature. Proteins were revealed by chemiluminescence with SuperSignal West Femto Chemiluminescent (Pierce).

### Data analysis

**Fig. 1:**
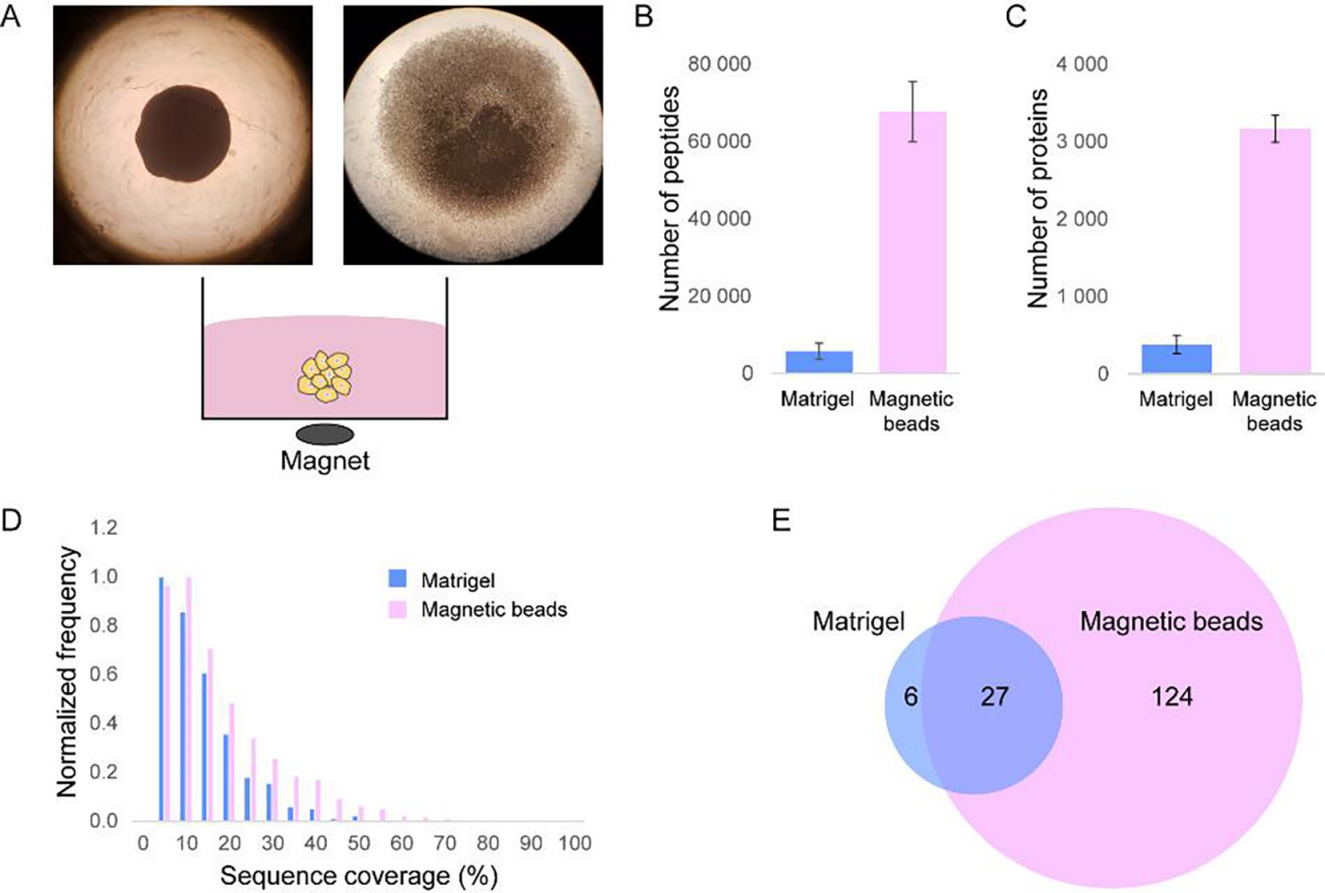
MS-based proteomic analyses of scaffold-free 3D cell cultures. (A) Images of NHF (left) and SCC13 (right) 3D spheroids generated by magnetic 3D bioprinting using NanoShuttle™-PL. A schematic representation of the cell culture system is shown below images. (B, C) Numbers of identified peptides (B) and proteins (C) by MS-based proteomics comparing Matrigel/collagen I- and magnetic bead-based 3D cell cultures. Shown are average numbers of 3 biological replicates. Error bars indicate standard deviation. (D) Histograms of binned sequence coverages of identified proteins comparing the indicated cell culture approaches. (E) Venn diagram comparing numbers of identified matrisome proteins by the different cell culture approaches. Sizes of circles indicate magnitude of identification rates.

**Fig. 2:**
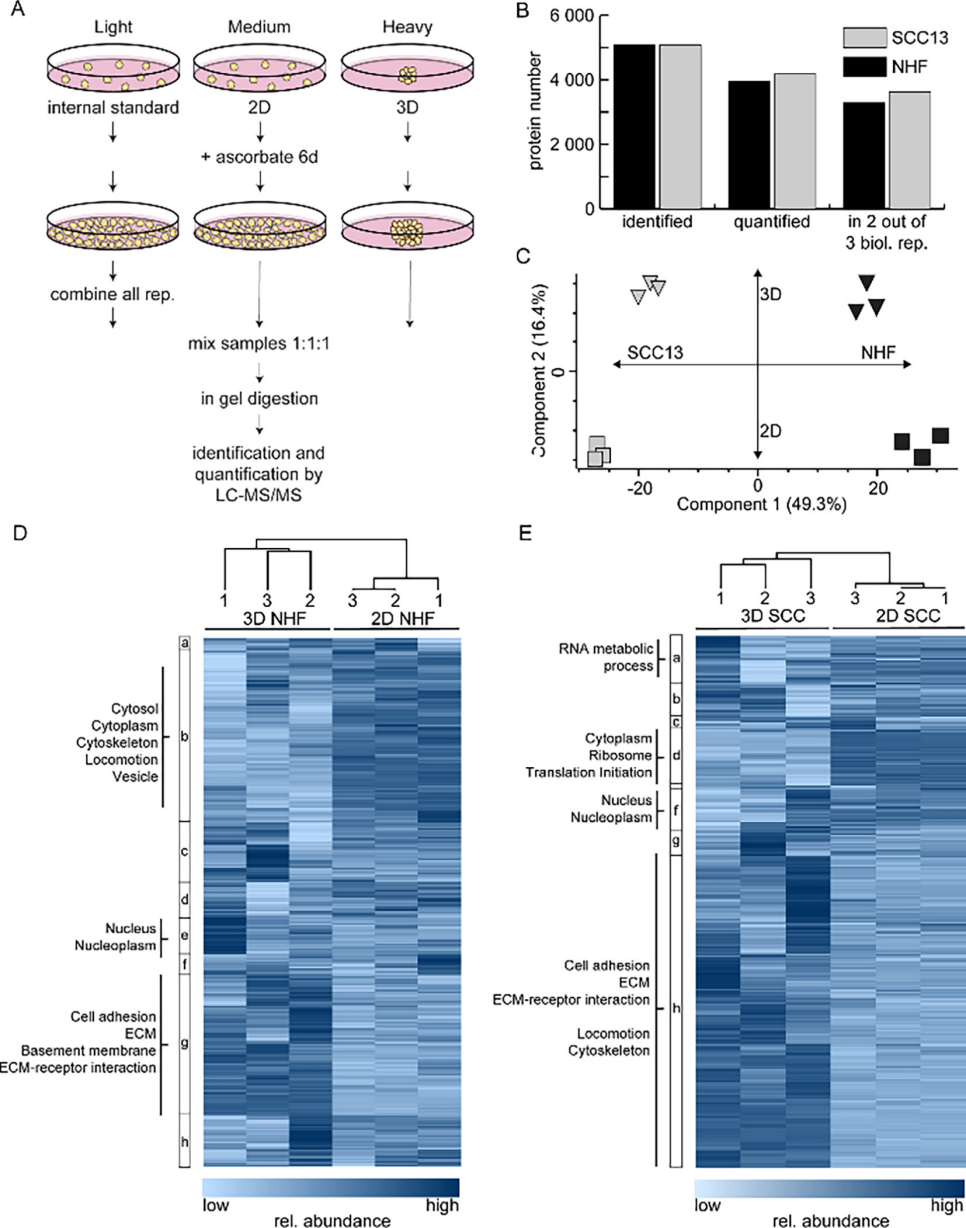
Regulation of the proteome in scaffold-free 3D cell cultures. (A) Super-SILAC-based proteomic workflow. (B) Numbers of identified and quantified proteins. (C) Principle component analysis of all analysed samples. Three biological replicates each of 2D (squares) and 3D (triangles) NHF (black) and SCC13 (grey), respectively, were performed. (D, E) Hierarchical clustering of samples and proteins using log2 and z-transformed protein ratios as input. 2D and 3D protein abundances were compared to the Super-SILAC spike-in, each colored line representing one protein. Protein clusters are numbered a–h from top to bottom. Examples of representative, enriched GO terms of proteins in respective clusters are listed (FDR < 0.05, BH corrected).

**Fig. 3:**
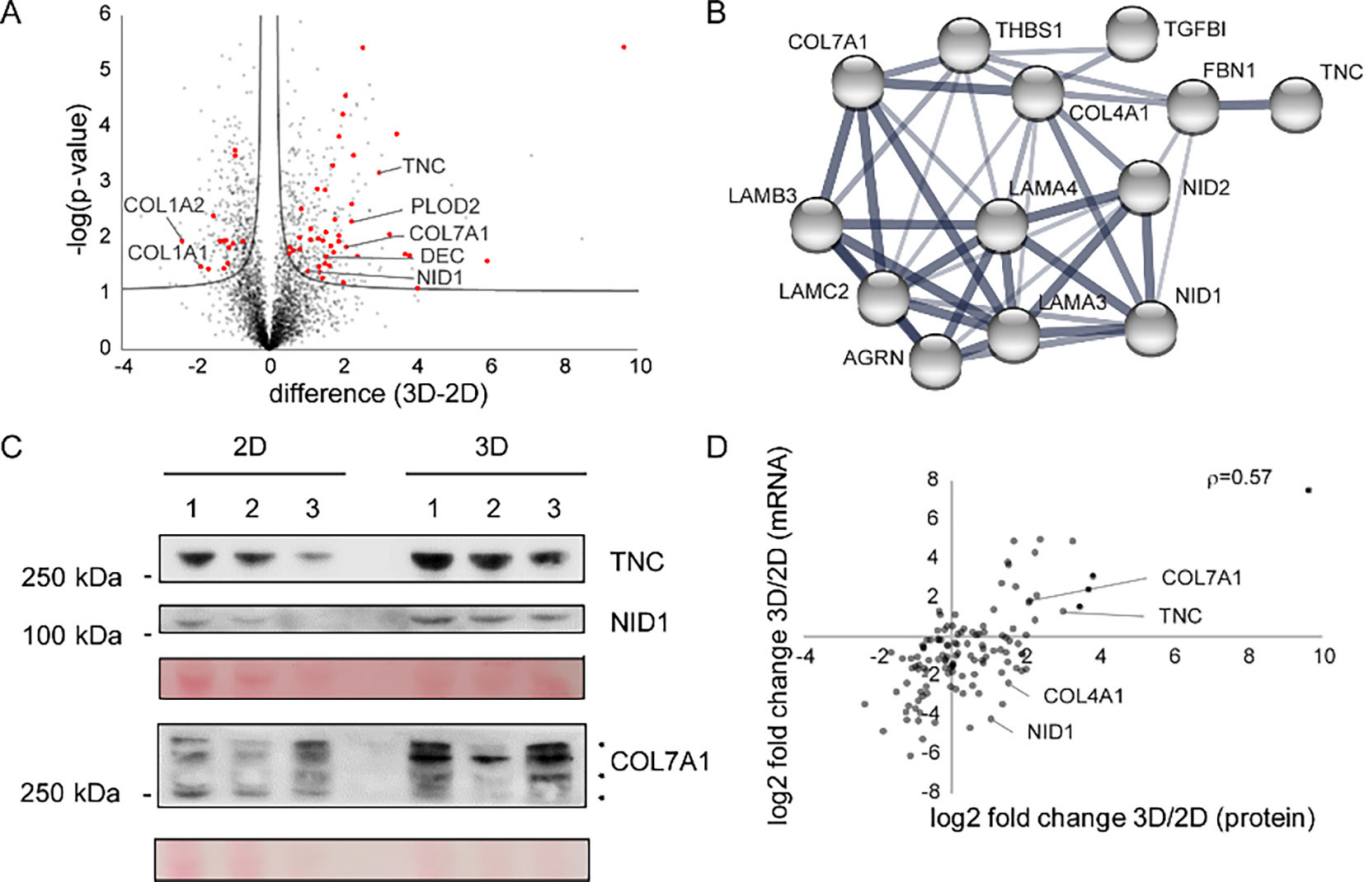
Regulation of the matrisome in scaffold-free 3D cultures. (A) Volcano plot comparing NHF protein abundances of cells grown in 3D and 2D. Solid lines indicate significantly changed proteins (FDR < 0.05, S0 = 0.1). Matrisome proteins are marked in red. (B) Protein-protein interaction network by STRING DB of significantly altered matrisome proteins linked to basement membrane biology [35]. Nodes indicate proteins, thickness of edges indicates confidence/strength of data supporting indicated interactions. (C) Western blot analysis of selected proteins confirms quantitative MS data. Ponceau staining was used as loading control. *: unspecific background bands. (D) mRNA-protein correlation analysis. Log2 transformed ratios of mRNA and protein abundances of matrisome constituents isolated from NHF grown in 3D and 2D are shown. *ρ* = Spearman's rank correlation coefficient. (For interpretation of the references to colour in this figure legend, the reader is referred to the web version of this article.)

For Fig. 1, published data of NHF grown in Matrigel were calculated with data generated in this study [9]. For Fig. 2, Fig. 3, NHF and SCC13 data were calculated and analysed using the Perseus software version 1.6.2.10 [23]. Normalized 3D/spike and 2D/spike ratios were log_2_ transformed. For principal component analysis (PCA), only proteins with valid values in all replicates were considered. For hierarchical clustering, ratios were normalized using z-score. For volcano plots, only proteins with 2 valid values out of 3 replicates were considered. The following settings were used: 3D on the right, 2D on the left, *t*-test on both sides with FDR < 0.05.

### RNA sequencing

RNA quality score (RIN^e^) was checked using the RNA ScreenTape System (Agilent), following the manufacturer's protocol. RNA sequencing was performed with 50 cycles of paired-end reads (NGS Sequencing Platform, Bern). QC was performed with FastQC v0.11.7 (https://www.bioinformatics.babraham.ac.uk/projects/fastqc/) and reads were cleaned using fastpv0.19.5 (https://doi.org/10.1093/bioinformatics/bty560) with a special focus on removing polyG trails and keeping only full-length reads (50 bp). The Human genome GRCh38.p13 from ENSEMBL [24] was indexed for STARv2.5.3a [25]. The cleaned reads were remapped to genes for each sample with STAR using ENSEMBL annotation information (Homo_sapiens.GRCh38.98.gtf). Gene expression quantified by RNA sequencing was matched to respective protein abundances generated by mass spectrometry by matching Gene Symbols.

Respective raw data are deposited at the European Nucleotide Archive [26], accession number: PRJEB43316, unique name: ena-STUDY-SIB-25-02-2021-12:20:32:813-1112.

## Results and discussion

### Increased protein identifications using scaffold-free 3D cell culture systems

Protein-containing scaffolds that support 3D cell culture inevitably interfere with MS-based proteomic mapping of cell-produced proteins. As an alternative, MS-compatible approach, we studied scaffold-free 3D cell cultures based on magnetic 3D bioprinting using NanoShuttle™-PL and compared protein identification rates of these to well-established 3D cell cultures based on a mix of Matrigel™ and collagen I (Fig. 1A) [9]. Importantly, as we were interested in ECM proteins, we did not purify cells from Matrigel cultures, but used a metabolic labelling approach based on Stable Isotope Labelling by Amino acids in Cell culture (SILAC) to discriminate endogenous, deposited ECM proteins produced by cells from exogenous scaffold proteins [9]. Comparing proteomes of 3D cultured primary normal human fibroblasts (NHF) isolated from skin, the scaffold-free approach yielded significant higher numbers in identified peptides and proteins using standard shotgun proteomics than the Matrigel/collagen I-based approach (Fig. 1B, C). In agreement, also the average sequence coverage of identified proteins increased from 10.9% to 15.4% using the scaffold-free approach (Fig. 1D). Importantly, matrisome identifications could be increased more than 4.5-fold using the scaffold-free method (Fig. 1E) [27]. Thus, to study effects of or in 3D cell cultures by MS-based proteomics, scaffold-free approaches based on magnetic 3D bioprinting yield significantly higher numbers and coverage of intracellular and matrisome proteins, supporting more detailed analyses of proteome dynamics.

### Scaffold-free 3D cell culture conditions lead to dramatic proteome changes

Cells grown in 3D scaffolds upregulate proteins that are important for cell-cell and cell-matrix interactions [9], presumably to ensure a proper architecture of the macroscopic 3D structure. However, depending on used scaffolds produced ECMs might differ and it is still under debate how well these systems recapitulate *in vivo* situations [10]. Also, in scaffold-free systems based on magnetic nanoparticles, cells produce more elaborate ECMs than 2D systems [28]. However, analyses were mainly performed for single proteins using immunofluorescent imaging or western blot analysis. To get an unbiased picture of proteome regulation due to 3D scaffold-free growth, we performed expression proteomics based on magnetic 3D bioprinting in combination with quantitative, SILAC-based MS (Fig. 2A). Briefly, the epidermal squamous cell carcinoma line SCC13 and primary NHF were metabolically labelled for one and two weeks, respectively, due to differential proliferation rates and to ensure full label incorporation. NHF had a doubling time of 2.1 days and SCC13 of 1 day. Next, cells were kept for 6 days in conventional 2D or NanoShuttle™-PL-based 3D cultures. In this period, ascorbate was added to support proper folding and secretion of collagens [29]. Using a light-labelled Super-SILAC spike-in containing both cell types, we were able to compare proteomes of 2D with 3D cultures between both cell types [18]. Samples were mixed 1:1:1 on protein level and analysed by quantitative liquid-chromatography (LC)–tandem MS (MS/MS). We identified more than 5000 proteins per cell type in three biological replicates each and could reproducibly quantify more than 3200 proteins for NHF, and more than 3600 proteins for SCC13 cells in minimally two out of three replicates (Fig. 2B, Supplementary Tables S1 and S2). Using a principal component analysis (PCA), cell types and culture conditions could be clearly discriminated (Fig. 2C). Interestingly, 3D culture conditions appeared to induce cell-type independent changes indicating that cells at least partially respond with a common gene expression program to a 3D environment. Comparing proteomic data generated *in vitro* in this study with published proteomic fingerprints of *in vivo* samples of 63 squamous cell lung and 49 head-and-neck carcinomas indicates that the proteomes of 3D cell cultures are indeed more similar to those of primary tissue samples than the proteomes of 2D cell cultures (Supplementary Fig. S1) [30].

To get an overview of protein regulation, we performed hierarchical clustering (Fig. 2D, E). 2D and 3D samples of a specific cell type nicely separated, indicating global changes due to cell culture conditions. Proteins carrying gene ontology (GO) terms “ECM”, “ECM-receptor interaction” and “cell adhesion” were enriched in 3D conditions in both cell types, indicating a cell type independent, common response to 3D. In 2D, proteomes between cell types differed more, as shown by the PCA (Fig. 2C). NHF exhibited higher abundances of proteins linked to cytoskeleton and cell migration suggesting a higher motility of NHF in 2D compared to 3D, as described for various cell types [31], [32] (Fig. 2D). Interestingly, these terms were associated to SCC13 cells in 3D. Whether epidermal cancer cells are more motile in 3D compared to 2D in the used model will have to be determined in future studies. In 2D, proteins linked to protein biosynthesis were more abundant in SCC13 cells (Fig. 2E).

Taken together, increased abundances of extracellular and cell adhesion proteins observed in 3D are not limited to protein containing scaffold-based cultures, in which exogenous proteins might stimulate or inhibit cells to secrete respective biomolecules. The ECM appears rather a result of the 3D assembly. Also, scaffold-free 3D cell culture conditions lead to profound changes of the matrisome, likely better reflecting *in vivo* observations and warranting further analyses.

### Scaffold-free 3D cell cultures increase matrisome deposition

Focusing our further analyses on the NHF matrisome, we observed that 44 out of a total of 133 quantified matrisome proteins were significantly enriched in 3D compared to 2D cultures and only 14 matrisome proteins were more abundant in 2D (Fig. 3A, FDR < 0.05, Supplementary Table S1). Especially the high abundance of both chains of the major interstitial collagen I in 2D was rather surprising. In 3D, proteins involved in collagen maturation were more abundant, like lysyl hydroxylases PLOD1 and PLOD2 [33], and the small leucine-rich proteoglycan decorin, which supports amongst others collagen fibrillogenesis [34] (Supplementary Table S1). Interestingly, all significantly regulated proteins linked to basement membrane biology were upregulated in 3D and we could construct a protein-protein interaction network of 14 members (Fig. 3B). The majority of these proteins were also significantly upregulated in SCC13 cells in 3D (Supplementary Fig. S2) further supporting the conclusion that 3D culture conditions induce cell-type independent changes indicative of a common gene expression program in response to a 3D environment (Fig. 2C). The increased abundances of collagens VII, nidogen 1 and tenascin C detected by quantitative MS could also be confirmed by western blot (Fig. 3C). Thus, 3D cultures appear to generate a more complex ECM, better reflecting *in vivo* tissue architecture.

To identify which matrisome proteins were differentially regulated on mRNA and protein level compared to protein level only indicating additional, more complex posttranscriptional regulation, we performed high-throughput sequencing using RNAseq of the same samples used for proteomic analyses. Matrisome mRNA and protein abundance differences due to culture conditions correlated with a *Spearman's* rank correlation coefficient *ρ* of 0.57 (Fig. 3D), which is higher than the entire dataset's correlation (Supplementary Fig. S3). This indicates that compared to intracellular proteins, matrisome protein abundance can be better predicted using mRNA data, which is a bit of a surprise keeping in mind the additional layer of regulation by protein secretion. This could indicate that matrisome proteins are generally more stable than intracellular proteins and that the effects of protein turnover are limited in the extracellular environment. Nevertheless, the abundance of roughly one third of matrisome proteins cannot be robustly predicted using mRNA abundance measurements. Interestingly, proteins from the same sub-compartment exhibit differential regulatory mechanisms. E.g. whereas collagen VII mRNA and protein abundance differences between 2D and 3D correlated well, this was not the case for nidogen-1, which is often found coregulated on protein level with collagen VII. In contrast, tenascin C, which is often opposingly regulated on protein level compared to collagen VII, mirrors collagen VII mRNA and protein changes [36]. Thus, it appears that it is difficult to formulate general rules for mechanisms governing protein abundances of matrisome proteins in 3D and that these have to be studied individually for each protein, even if these are known to interact as highlighted by the differential regulations of collagens IV and VII (Fig. 3D).

Taken together, magnetic bioprinting as an enabling tool supports detailed proteomic analyses of 3D cell cultures. Due to the absence of non-human, protein containing scaffolds, cells and their endogenous ECM can be holistically analysed allowing detailed insights into proteome dynamics supporting 3D architecture underlying tissue organization. In combination with drug testing, these methods promise exciting insights into the regulation of the ECM, its role in regulating tissue homeostasis and its contribution to disease.

## CRediT authorship contribution statement

**Bich Vu:** Investigation, Methodology, Visualization, Writing - review & editing. **Glauco R. Souza:** Writing - review & editing. **Jörn Dengjel:** Conceptualization, Visualization, Writing - review & editing, Funding acquisition, Resources, Supervision.

## Declaration of Competing Interest

The authors declare the following financial interests/personal relationships which may be considered as potential competing interests: Glauco R. Souza is employed by Greiner Bio-One GmbH which produces NanoShuttle™-PL.
