# Supplementary material for: Scaffold-free 3D cell culture of primary skin fibroblasts induces profound changes of the matrisome
Source: Matrix Biol Plus. 2021 May 12;11:100066. doi: 10.1016/j.mbplus.2021.100066 (PMC8377039; doi:10.1016/j.mbplus.2021.100066)
Supplement: Supplementary data 1 [file mmc1.docx]

**Supplementary Figure S1**

**
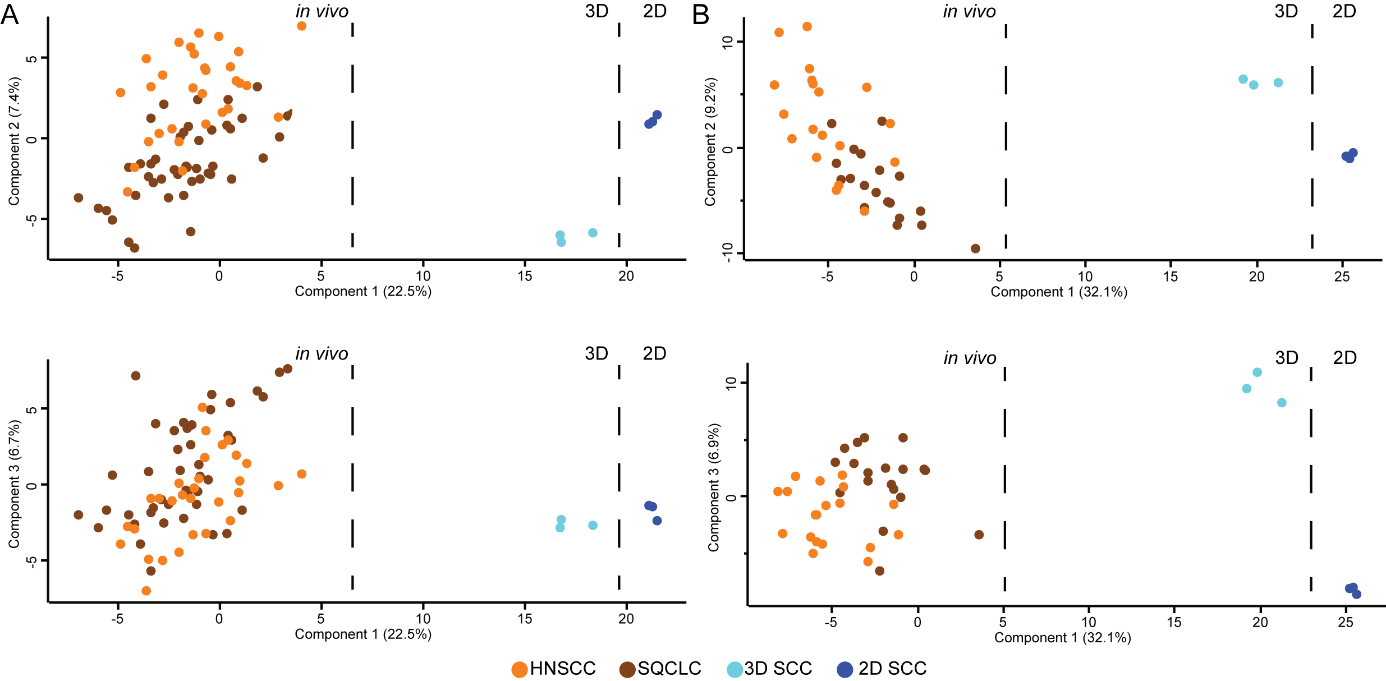
**

**Supplementary Figure S1 : Principle component analyses comparing *in vivo* and *in vitro* expression proteomics datasets.** Comparison of published expression proteomics data of (A) 44 squamous cell lung carcinomas (SQCLC) and 30 head-and-neck squamous cell carcinomas (HNSCC), and (B) 19 SQCLC and HNSCC each, with data of 3D and 2D *in vitro* cell cultures generated in this study indicates that 3D cultures are more similar to primary samples than 2D cultures (30).

**Supplementary Figure S2 :**

**
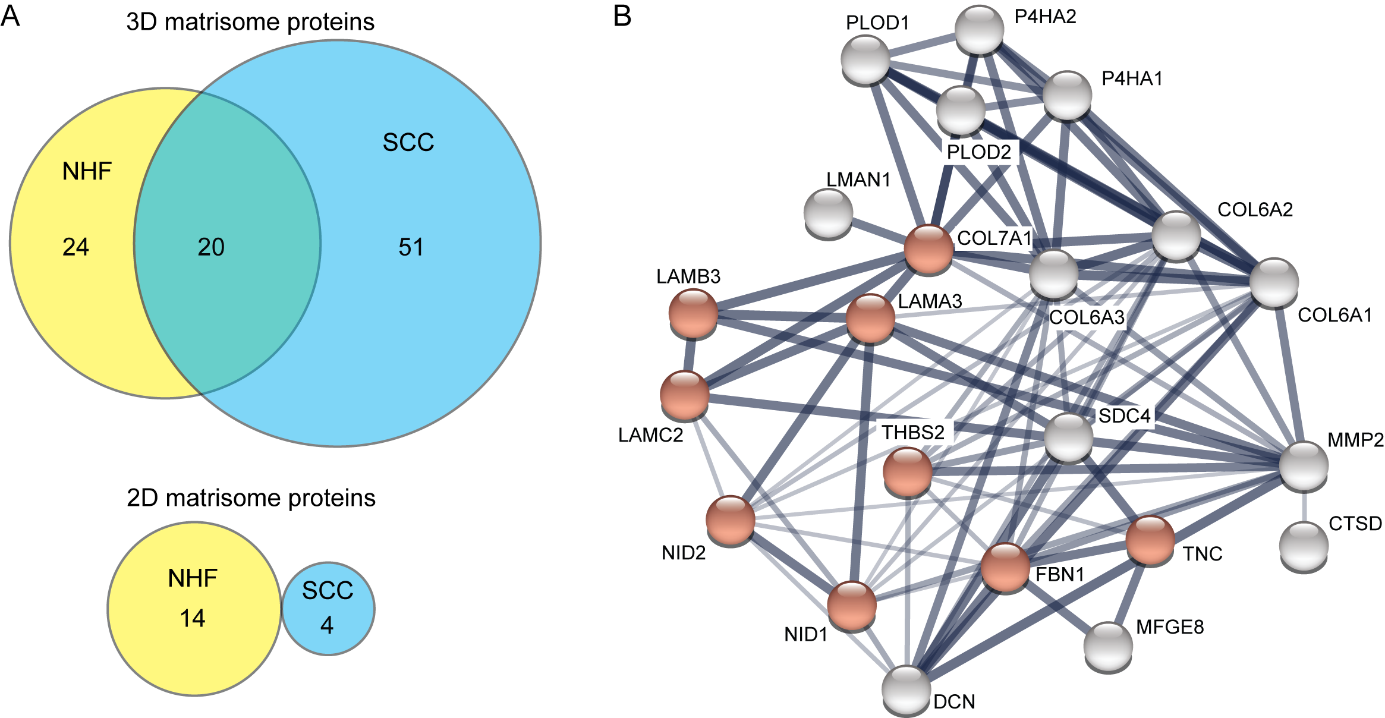
**

**Supplementary Figure S2 : Comparison of matrisome changes between SCC13 cells and NHF due to culture conditions. (A)** Venn diagrams indicating numbers and overlap of significantly regulated matrisome proteins in SCC13 cells and NHF. Note that 3D culture conditions lead to the identification of more and commonly regulated matrisome proteins (n=3, FDR<0.05, S0=0.1). Sizes of circles indicate numbers of quantified proteins. **(B)** Protein-protein interaction network by STRING DB of commonly significantly altered matrisome proteins comparing SCC13 cells and NHF (35). Proteins linked to basement membrane biology are highlighted in red. Nodes indicate proteins, thickness of edges indicates confidence/strength of data supporting indicated interactions.

**Supplementary Figure S3:**

**
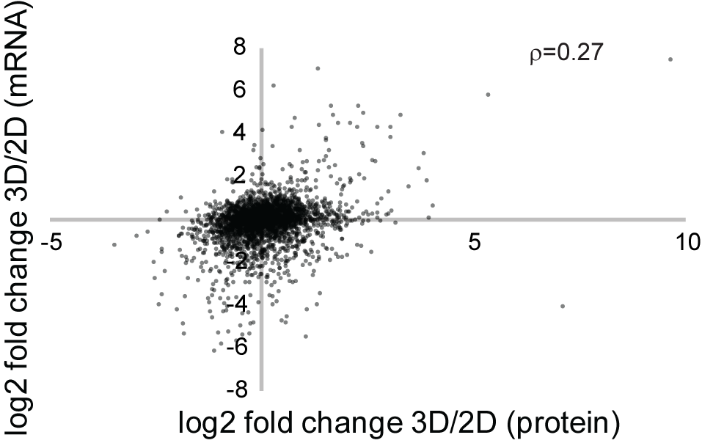
**

**Supplementary Figure S3 : Global mRNA-protein correlation analysis.** Log2 transformed ratios of abundances of mRNAs and proteins isolated from NHF grown in 3D and 2D are shown. ρ= Spearman's rank correlation coefficient.
